# Financial Distress and Psychological Well-Being During the COVID-19 Pandemic

**DOI:** 10.3389/ijph.2022.1604591

**Published:** 2022-08-25

**Authors:** Florencia Borrescio-Higa, Federico Droller, Patricio Valenzuela

**Affiliations:** ^1^ Business School, Universidad Adolfo Ibañez, Santiago, Chile; ^2^ Departamento de Economía, Universidad de Santiago de Chile, Santiago, Chile; ^3^ Facultad de Ingeniería y Ciencias Aplicadas, Universidad de los Andes, Santiago, Chile

**Keywords:** mental health, COVID-19, well-being, financial distress, savings

## Abstract

**Objective:** We examine the impact of financial distress caused by the COVID-19 pandemic on mental health and psychological well-being.

**Methods:** We analyze cross-sectional survey data (*n* = 2,545) from the Life during Pandemic study in Chile. We estimate linear probability models to analyze the relationship between economic fragility, financial distress, and psychological well-being.

**Results:** Our findings show unemployment and income loss are highly predictive of experiencing a range of financial problems, such as a lack of savings, as well as difficulties paying bills, consumer debt, and mortgage loans. In turn, financial distress leads to a higher prevalence of poor well-being and mental health deterioration, and sleep problems.

**Conclusion:** Expansion of mental health assistance services are needed, as new diagnosis of mental health conditions has increased, but treatment has not, pointing to a barrier in the access to some mental health care services during the pandemic. Policies designed with the objective of improving financial education are necessary to increase precautionary savings and financial resilience, and alleviate the psychological burden of debt in the future.

## Introduction

The COVID-19 pandemic produced an unprecedented and long-lasting shock to households around the world. Most countries have seen severe declines in income and higher rates of unemployment [1, 2]. In line with economic concerns and food insecurity, mental health and well-being deteriorated significantly during the pandemic [3–7].

In this paper we investigate the economic effects of the COVID-19 pandemic and the role of financial distress in the mental health and well-being of individuals, using a novel dataset from the *Vida en Pandemia* (Life during Pandemic) survey in Chile, which combines labor outcomes, financial fragility, and mental health information. We hypothesize that pandemic-induced unemployment and loss of income increases financial hardship, which in turn increases vulnerability to mental health difficulties and illnesses.

Typically, measures of financial hardship include difficulty paying bills, purchasing basic goods such as food and clothing, and affording suitable housing, utilities, health care, and transportation [8]. Personal debt is closely related to common mental disorders, and financial hardship is strongly related to a higher risk of depression, relative to other measures of income and socio-economic status [9–12]. In particular, the association between debt and depressive symptoms seems to be driven by consumer credit or late mortgage payments [13].

Symptoms of depression increased with job loss among older workers during the Great Recession, and wealth has a mitigating effect in this relationship [14]. Unemployment is also closely related to financial hardship, because income loss due to unemployment is a good predictor of the inability to pay a mortgage or credit loan [15–17]. Job loss is also related to mortality [18], cardiovascular diseases [19–21], and subjective well-being and mental health [22, 23]. Stock market declines are related to increases in hospital admissions for psychological conditions [24] and declines in subjective measures of mental health [25].

Recent studies have documented the effect of the COVID-19 pandemic on mental health and well-being in different countries and circumstances. There are several reviews on mental health early consequences of the COVID-19 pandemic [26–28]. There is evidence of the worsening in psychological health in Canada and how economic concerns (including food insecurity) play a role in explaining poor mental health at the onset of the pandemic [5]. A study on subjective well-being reports that individuals who suffered a drop in household income during the pandemic experienced a large decline in overall life satisfaction [29]. Another study documents deterioration in mental health in United Kingdom, which is heterogeneous across ethnicity and gender [4]. There is also evidence showing an increase in psychological distress and mental health disorders in low and middle income countries [30]. Further, financial worries of self-employed workers are associated with higher mental distress during the pandemic [31]. Google Trends data for Latin American countries show searches for words associated with mild mental health disorders including insomnia, stress, and anxiety increased during the COVID-19 stay-at-home orders [32]. Related to our study, a recent paper shows increasing stress and worsening mental health associated to economic vulnerability in Spain, Italy and the United Kingdom [33].

Chile provides an interesting case study as a country that has experienced significant economic growth over the past few decades, but where high levels of income inequality and informality are still pervasive [34]. Poor economic outcomes caused by the COVID-19 pandemic gave rise to increased household financial fragility. First, lockdowns were implemented locally and announced weekly, after the first case was announced in early March 2020 [35]. Overall economic activity in Chile dropped significantly in 2020, and lockdowns have been strongly associated with a decrease in income, an increase in unemployment, and an increase in household debt [36, 37]. Unemployment rates increased from 8.2% in the first quarter of 2020 to 12.9% at the time of the survey [38]. Survey data show that over half of respondents saw a decrease in household income (37% due to unemployment) and an increase in household debt by 39% relative to pre-pandemic levels [39]. Critically, household debt had been growing in the past several decades, reaching a historical maximum of 76.4% of annual disposable income relative to the second quarter of 2019 [13, 40]. Starting in March 2020, the Chilean government implemented a series of policies to alleviate the effects of the pandemic on household. The main measures include a voucher for poor families, and the deferral of utility payments and certain taxes. Commercial banks voluntarily implemented the deferral of mortgage and commercial loan payments, selecting customers who had no arrears prior to March. A detailed analysis of the set of policies implemented argues that tax relief had a relatively small impact, and that the debt-deferral benefits were concentrated in a few highly indebted and richer agents [41].

The paper’s major findings indicate unemployment and income loss are highly predictive of experiencing a range of financial problems, such as a lack of savings and difficulty paying bills, consumer debt, and mortgage loans. In turn, financial distress leads to a higher prevalence of mental health and well-being complaints, including feelings of depression, anxiety, and sleep problems. Our main findings are robust to alternative measures of financial distress, mental health and well-being, and potential endogeneity bias.

This study contributes to the new body of research that explores how economic and financial fragility is one of the main stressors contributing to widespread emotional stress during the pandemic, and how some specific groups are more vulnerable than others to the psychological effects of pandemics. The understanding of the nexus between financial fragility and mental health is important, and we add evidence that is relatively scarce outside developed countries. Our results suggest the effects of the COVID-19 pandemic has worrying implications for those in more vulnerable economic and financial situations.

## Methods

### Data

We use data from the *Vida en Pandemia* survey in Chile [42]. The survey was implemented 13–17 July 2020, through phone calls (from a large database of phone numbers obtained from the merge of administrative and private databases), with 2,545 adult respondents. The survey was designed to be balanced in terms of age, gender, and municipality of residence, to render representativeness across age, gender, and socioeconomic status. Because the data are deidentified, this study was not considered human-subjects research.

The survey contains information on basic demographic variables of respondents, including age, gender, educational level, socioeconomic characteristics, and municipality of residence. Further, it contains detailed information on employment, economic hardship, financial health, living arrangements, and a series of measures on self-reported well-being.

### Main Variables

The main variables in this study are divided into five groups: financial distress, economic fragility, psychological well-being and emotions, utilization of mental health-care services, and conflict within the household. The measures of financial distress include an indicator variable for difficulty paying mortgage loans (“During the pandemic, have you had difficulties paying your mortgage monthly payment? 1 = yes, 0 = no) and an indicator variable for difficulty paying consumer debt (“During the pandemic, have you had difficulties to pay your consumer debt on time? 1 = yes, 0 = no). We also construct a measure from 0 to 4 regarding whether the individual has problems paying for basic goods and services: 1) basic goods, 2) medicine, 3) rent, and/or 4) school.

Savings are measured as the number of months the respondent believes that basic expenses can be afforded with savings if the main income source is lost. We create a lack of savings variable, that is an indicator that equals 1 if the individual reports that the household’s savings are below the sample median (3 months), and 0 otherwise. We measure economic fragility with indicator variables for unemployment (own or family member, separately) and income loss. The former is equal to 1 if the individual (or a family member) reports unemployment after March 2020 as a direct consequence of the pandemic, and 0 otherwise. The indicator for income loss is equal to 1 if the income reported in May 2020 is lower than the income reported for February 2020, and 0 otherwise.

Measures of psychological subjective well-being include sleep problems (“During the last week, did you have sleeping problems?”), and negative emotions, such as distress, frustration, worry, and restlessness (“During the last 2 weeks, how often have you felt … ?”). Responses to these variables are categorized by frequency in a 4-point Likert scale (4 = very frequently, 3 = frequently, 2 = sometimes, 1 = never). We construct a dichotomous variable for each measure, that is equal to 1 if the individual reports experiencing the abovementioned condition or feeling frequently or very frequently, and 0 otherwise. We create an indicator variable for poor well-being, based on a measure (“How would you describe your current well-being?” 5 = very good, 4 = good, 3 = neither good nor poor, 2 = poor, 1 = very poor), where the indicator is equal to 1 if the individual reports feeling poor or very poor well-being, and 0 otherwise. We also include an indicator for well-being deterioration that is equal to 1 if the individual reports that her well-being or mental health has worsened relative to February (before the pandemic), and 0 otherwise [“Would you say your well-being or mental health has (3 = improved, 2 = is the same, 1 = worsened) compared to before March 2020”]. Note that although the survey design is cross-sectional, this variable allows us to capture the change in well-being as reported by individuals.

Utilization of mental health-care services is measured with a set of indicator variables that capture a new diagnosis, new treatment, and/or new medication (for a mental health condition), after the start of the pandemic (1 = yes, 0 = no). Finally, we consider two variables that measure conflict within the household: an indicator for higher frequency of conflicts relative to before the pandemic, and a variable that measures severity of conflicts. Severity of the conflicts is a variable that goes from 0 to 5, where 0 indicates there is not an increase in conflicts, 1 indicates an increase in conflicts of low intensity, and 5 indicates an increase in conflicts of high intensity.

### Empirical Strategy

First, we analyze the relationship between economic fragility and financial distress, following a linear probability model (LPM):

$$\mathrm{financial}\_\mathrm{distres}s_{\mathrm{ir}}=\alpha +\beta_{1}econ\_\mathrm{fragilit}y_{\mathrm{ir}}+X_{\mathrm{ir}}^{\prime }\beta_{2}+A_{r}+\varepsilon_{\mathrm{ir}},$$

where $\mathrm{financial}\_{\mathrm{distress}}_{ir}$ is a dependent variable of interest of individual i in region r (difficulty paying mortgage loans, difficulty paying consumer loans, difficulty paying for basic goods and services, and lack of savings), and $econ\_{\mathrm{fragility}}_{i}$ represents own (or family) unemployment, or income loss. We include a set of control variables in the vector $X_{i}$ . Control variables include the following: an indicator for female, age-category dummies (18–24, 25–34, 35–44, 45–54, 55–64, and over 65), an indicator for household head, education-achievement indicators (complete or incomplete primary, secondary, technical, bachelor’s, and master’s level or more), indicators for the presence of young children (under the age of 12) in the household, migrant status, and pre-pandemic household income (expressed in natural logarithm). All regressions include a set of region indicators ( $A_{r}$ ) to account for fixed characteristics at the region level. Standard errors are robust to heteroskedasticity.

We then analyze the relationship between financial distress and psychological well-being, according to the following equation:

$$y_{ir}=\alpha +\beta_{1}\mathrm{financial}\_\mathrm{distres}s_{\mathrm{ir}}+\beta_{2}{\mathrm{unemployed}}_{\mathrm{ir}}+X_{\mathrm{ir}}^{\prime }\beta_{3}+A_{r}+\varepsilon_{\mathrm{ir}},$$

where $y_{ir}$ is an outcome variable of individual i in region r (poor well-being, sleep problems, well-being deterioration, new diagnosis of mental health condition, new treatment, and new medication for mental health condition). We use the same set of controls and include own unemployment. We conduct linear probability models because estimating logit (probit) models with many dummy variables (e.g., a set of indicators by region) may introduce an incidental parameter problem. However, all our findings are robust to estimations *via* logit/probit models (results available upon request).

## Results

### Descriptive Statistics

Supplementary Table A1 in the appendix reports descriptive statistics for all outcome variables, and Supplementary Table A2 reports descriptive statistics for individual characteristics. In the final sample employed in our baseline regression, 47% of the respondents are female, 7% are migrants, 57% have children, 47% have a college degree, and 22% experienced unemployment (own or family member) during the pandemic.

Supplementary Table A3 in the appendix reports correlations between mental health measures and individual characteristics. Women and younger adults experience on average worse mental health, facing higher rates of poor well-being, sleep problems and mental health deterioration. Being unemployed has a negative effect on mental health. Indicator variables for household head and for education levels appear not to be associated with worse mental health in this sample and income has a weak negative correlation with poor well-being.

### Financial Distress and Economic Fragility

**FIGURE 1:**
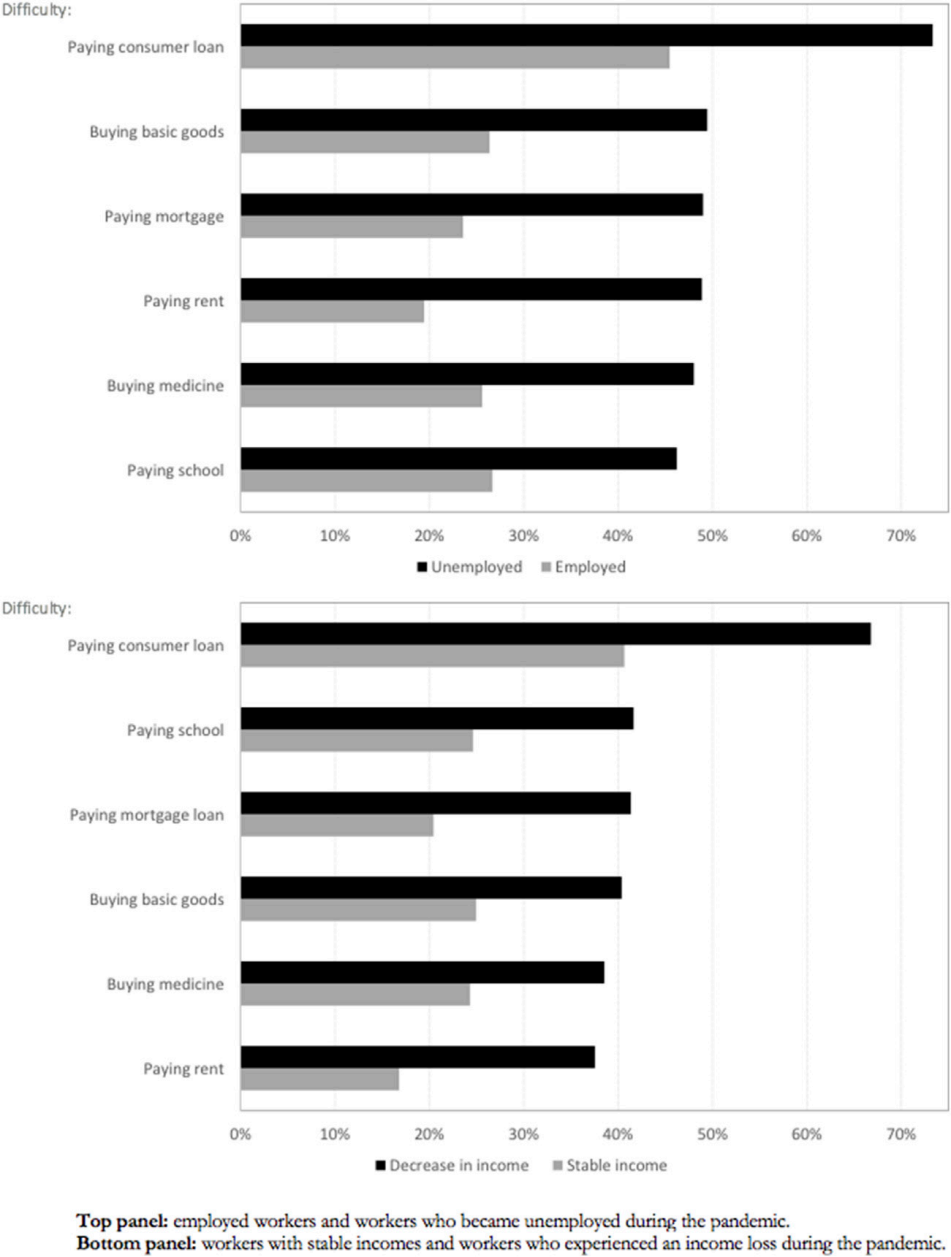
Economic fragility and financial distress (Chile, 2020).

Figure 1 shows the proportion of individuals facing financial problems by their labor status, we compare employed workers with workers who became unemployed, and workers with stable income with workers who experienced an income loss, during the pandemic. As expected, the figure shows that workers who became unemployed or suffered an income loss were more likely to face problems paying their debts and bills.

Table 1 explores the relationship between economic hardship and financial distress, which capture individuals reporting not being able to meet financial obligations during the pandemic period, after controlling for a comprehensive set of variables. Individuals who became unemployed or who experienced an income drop are more likely to experience financial problems, such as problems paying their mortgages [columns (1) and (2)], consumer debt [columns (3) and (4)], and difficulty paying basic goods and services [columns (5) and (6)]. They also have less savings to face future financial obligations.

**TABLE 1:** Financial distress and economic fragility (Chile, 2020).

Difficulty Paying	(1)	(2)	(3)	(4)	(5)	(6)	(7)	(8)
	Mortgage Loan		Consumer Debt		Basic Goods and Services		Lack of savings	
Female	−0.0510**	−0.0438*	0.0268	0.0092	−0.0361	−0.0525	0.0565***	0.0450**
	(0.0256)	(0.0264)	(0.0237)	(0.0235)	(0.0501)	(0.0510)	(0.0215)	(0.0216)
Age	−0.0018**	−0.0016*	−0.0030***	−0.0026***	−0.0076***	−0.0072***	−0.0050***	−0.0048***
	(0.0008)	(0.0008)	(0.0008)	(0.0008)	(0.0016)	(0.0016)	(0.0007)	(0.0007)
Migrant	0.0822	0.0910*	0.0236	0.0294	0.1110	0.0879	0.0705*	0.0437
	(0.0524)	(0.0499)	(0.0486)	(0.0485)	(0.0991)	(0.1050)	(0.0395)	(0.0402)
Children in household	0.0683**	0.0873***	0.0755***	0.0860***	0.1700***	0.2030***	0.0788***	0.0916***
	(0.0288)	(0.0294)	(0.0257)	(0.0255)	(0.0582)	(0.0583)	(0.0235)	(0.0234)
Household head	−0.0225	−0.0069	−0.0014	0.0039	0.0094	0.0120	0.0327	0.0309
	(0.0269)	(0.0276)	(0.0251)	(0.0251)	(0.0535)	(0.0538)	(0.0228)	(0.0230)
ln (1 + Income pre-pandemic)	−0.0221***	−0.0918***	−0.0308***	−0.1640***	−0.0850***	−0.3810***	−0.0286***	−0.1380***
	(0.0067)	(0.0166)	(0.0053)	(0.0141)	(0.0133)	(0.0315)	(0.0049)	(0.0145)
Unemployment	0.2440***		0.2300***		0.6010***		0.0539**	
	(0.0360)		(0.0283)		(0.0666)		(0.0258)	
Family member unemployment	0.2270***		0.1740***		0.4930***		0.0873***	
	(0.0333)		(0.0271)		(0.0614)		(0.0244)	
Income drop		0.2140***		0.2710***		0.5080***		0.0803***
		(0.0277)		(0.0232)		(0.0524)		(0.0216)
Observations	1,201	1,182	1,736	1,705	1,919	1,881	1,947	1,908
R-squared	0.149	0.120	0.149	0.191	0.191	0.185	0.125	0.152
Education level	YES	YES	YES	YES	YES	YES	YES	YES
Region fixed effects	YES	YES	YES	YES	YES	YES	YES	YES

Note: This table reports estimates from a linear probability model (LPM) of the probability of experiencing a range of financial problems against the independent variables. The measures of financial distress include an indicator variable for difficulty paying mortgage loans and an indicator variable for difficulty paying consumer debt. We also construct a measure from 0 to 4 regarding whether the individual has problems paying for basic goods and services: 1) basic goods, 2) medicine, 3) rent, and/or 4) school. Finally, we use an indicator variable for lack of savings, that is equal to 1 if the number of months the respondent believes that basic expenses can be afforded with savings if the main income source is lost is below the sample median. All regressions control for education and region dummy variables. Heteroskedasticity-robust standard errors are in parentheses. ***, **, and * indicate significance at the 1%, 5%, and 10% levels, respectively.

The relationship between financial distress and unemployment or income drop is highly statistically significant and economically meaningful regardless of the model specification. Our estimates indicate unemployment increases the probability of having problems paying a mortgage loan or a consumer loan by roughly 23 and 24 percentage points, respectively, after controlling for pre-pandemic income, a comprehensive set of variables, education, and region fixed effects. Our estimates also suggest that, on average, unemployment increases roughly by 0.6 the number of different basic goods or services one may have problems paying for (i.e., basic goods, medicine, rent, and/or school). Finally, unemployment increases by 5 percentage points the probability of having insufficient savings to pay for basic needs for at least 3 months. Overall, results are qualitatively similar when we use an indicator of income drop as a measure of economic hardship.

Most of the coefficients associated with the control variables have the expected sign and are statistically significant. Older individuals have fewer financial problems and are less likely to have a lack of savings. Households with more children have more financial problems and less savings. Individuals with a higher pre-pandemic income are less likely to face any financial problem or lack savings. Finally, individuals with a family member who became unemployed also face a more difficult financial situation during the pandemic.

### Mental Health and Financial Distress

In Table 2 we explore the relationship between mental health and financial distress, controlling for a comprehensive set of variables. Results indicate individuals who experienced financial problems or had insufficient savings were more likely to experience poor well-being [columns (1) to (4)], sleep problems [columns (5) to (8)], and a deterioration of their well-being [columns (9) to (12)]. All our coefficients of interest are highly statistically significant and economically meaningful regardless of the model specification. For example, results in columns (9) and (10) indicate that having difficulty paying off a mortgage loan or consumer debt leads to a roughly 14-percentage-point increase in the probability of experiencing a deterioration of well-being. Column (11) indicates that having difficulty paying for one additional basic good or service leads to an 8-percentage-point increase in the probability of experiencing a deterioration of well-being. Finally, the results in column (12) suggest the lack of savings implies a 12-percentage-point higher probability of experiencing a deterioration of well-being.

**TABLE 2:** Mental health and financial distress (Chile, 2020).

	(1)	(2)	(3)	(4)	(5)	(6)	(7)	(8)	(9)	(10)	(11)	(12)
	Poor well-being				Sleep problems				Deterioration			
Unemployment	0.0779**	0.0259	0.0002	0.0606**	0.1300***	0.1020***	0.0966***	0.1420***	0.0607*	0.0666**	0.0630**	0.1050***
	(0.0375)	(0.0298)	(0.0271)	(0.0274)	(0.0375)	(0.0304)	(0.0281)	(0.0277)	(0.0365)	(0.0297)	(0.0273)	(0.0268)
Female	0.0601**	0.0643***	0.0605***	0.0576**	0.1090***	0.1130***	0.1060***	0.1040***	0.0826***	0.0880***	0.0852***	0.0842***
	(0.0284)	(0.0236)	(0.0221)	(0.0225)	(0.0291)	(0.0243)	(0.0228)	(0.0230)	(0.0295)	(0.0244)	(0.0228)	(0.0230)
Age	−0.0051***	−0.0046***	−0.0040***	−0.0046***	−0.0037***	−0.0037***	−0.0036***	−0.0039***	−0.0024**	−0.0029***	−0.0028***	−0.0029***
	(0.0009)	(0.0008)	(0.0007)	(0.0007)	(0.0010)	(0.0008)	(0.0007)	(0.0008)	(0.0010)	(0.0008)	(0.0008)	(0.0008)
Migrant	−0.0820	−0.1030**	−0.1180***	−0.1090***	−0.1070**	−0.1150**	−0.1070**	−0.1070**	−0.1830***	−0.1470***	−0.1700***	−0.1570***
	(0.0525)	(0.0430)	(0.0417)	(0.0410)	(0.0539)	(0.0473)	(0.0448)	(0.0433)	(0.0573)	(0.0494)	(0.0478)	(0.0471)
Children in household	−0.0161	−0.0076	−0.0161	−0.0036	0.0494	0.0538**	0.0496**	0.0558**	0.0607*	0.0674**	0.0580**	0.0582**
	(0.0307)	(0.0262)	(0.0244)	(0.0251)	(0.0317)	(0.0266)	(0.0251)	(0.0255)	(0.0313)	(0.0263)	(0.0248)	(0.0250)
Household head	0.0286	0.0411	0.0345	0.0374	−0.0256	−0.0216	−0.0235	−0.0214	−0.0126	−0.0097	−0.0087	−0.0093
	(0.0301)	(0.0251)	(0.0236)	(0.0239)	(0.0310)	(0.0262)	(0.0244)	(0.0246)	(0.0315)	(0.0261)	(0.0246)	(0.0246)
ln (1 + Income pre-pandemic)	−0.0155**	−0.0073	−0.0049	−0.0114**	−0.0030	0.0040	0.0073	0.0027	−0.0045	0.0000	0.0012	−0.0032
	(0.0073)	(0.0058)	(0.0055)	(0.0055)	(0.0071)	(0.0054)	(0.0050)	(0.0050)	(0.0073)	(0.0058)	(0.0054)	(0.0053)
Mortgage loans	0.0857***				0.1210***				0.1330***			
	(0.0326)				(0.0329)				(0.0322)			
Consumer debt		0.1500***				0.1300***				0.1460***		
		(0.0237)				(0.0248)				(0.0249)		
Basic goods and services			0.1040***				0.0841***				0.0818***	
			(0.0102)				(0.0105)				(0.0101)	
Lack of savings				0.0652***				0.0752***				0.1160***
				(0.0232)				(0.0240)				(0.0244)
Observations	1,201	1,736	1,919	1,947	1,201	1,736	1,919	1,947	1,201	1,736	1,919	1,947
R-squared	0.063	0.072	0.099	0.051	0.092	0.096	0.113	0.085	0.075	0.083	0.097	0.077
Education level	YES	YES	YES	YES	YES	YES	YES	YES	YES	YES	YES	YES
Region fixed effects	YES	YES	YES	YES	YES	YES	YES	YES	YES	YES	YES	YES

Note: This table reports estimates from a linear probability model (LPM) of the probability of experiencing a range of mental health problems against the independent variables. For mental health problems, we create an indicator variable equal to 1 if the individual reports feeling poor or very poor well-being. We also create a dummy variable for sleep problems during the last week. Finally, we include an indicator for well-being deterioration that is equal to 1 if the individual reports that her well-being or mental health has worsened relative to February (before the pandemic). All regressions control for education and region dummy variables. Heteroskedasticity-robust standard errors are in parentheses. ***, **, and * indicate significance at the 1%, 5%, and 10% levels, respectively.

Relatedly, in Supplementary Table A4 we examine whether financial problems are also associated with adults experiencing negative emotions such as distress, frustration, worry, and restlessness. All our measures of financial distress, including lack of savings, are associated with an increase of these negative emotions, ranging from 8 to 18 percentage points.

Table 3 examines whether financial distress affected utilization of mental health-care services during the COVID-19 pandemic, by analyzing whether individuals report having a new diagnosis and/or a new treatment of a mental health problem. On the one hand, the results reported in columns (2), (3), and (4) indicate individuals with fewer savings and financial problems (paying consumer debt and basic needs) are more likely to be newly diagnosed a mental health problem, but we do not find a statistically significant relationship between financial distress and mental-health treatment. Finally, columns (9) and (10) indicate individuals with problems paying off their mortgage and consumer debt are more likely to use mental health medication than individuals without those problems. We present a robustness check exercise in the Supplementary Material, where we re-estimate our baseline regressions using instrumental variables.

**TABLE 3:** Mental healthcare services and financial distress (Chile, 2020).

	(1)	(2)	(3)	(4)	(5)	(6)	(7)	(8)	(9)	(10)	(11)	(12)
	Diagnosis				Treatment				Medication			
Unemployment	0.0384**	0.0196	0.0210	0.0258*	0.0139	0.0022	0.0013	0.0029	0.0323*	0.0190	0.0247*	0.0271**
	(0.0188)	(0.0144)	(0.0134)	(0.0133)	(0.0165)	(0.0119)	(0.0111)	(0.0109)	(0.0187)	(0.0142)	(0.0135)	(0.0133)
Female	0.0261**	0.0196*	0.0229**	0.0212**	0.0362***	0.0251***	0.0270***	0.0271***	0.0302**	0.0182*	0.0180*	0.0165*
	(0.0131)	(0.0106)	(0.0099)	(0.0099)	(0.0127)	(0.0096)	(0.0092)	(0.0091)	(0.0130)	(0.0105)	(0.00987)	(0.0098)
Age	0.0000	0.0000	−0.0001	−0.0001	−0.0005	−0.0006*	−0.0006**	−0.0006**	0.0006	0.0002	0.0001	0.0001
	(0.0004)	(0.0003)	(0.0003)	(0.0003)	(0.0003)	(0.0003)	(0.0002)	(0.0002)	(0.0004)	(0.0003)	(0.0003)	(0.0003)
Migrant	−0.0144	−0.0051	−0.0102	−0.0099	0.0427	0.0233	0.0258	0.0261	−0.0311	−0.0084	−0.0113	−0.0112
	(0.0221)	(0.0195)	(0.0173)	(0.0169)	(0.0316)	(0.0229)	(0.0218)	(0.0211)	(0.0191)	(0.0193)	(0.0171)	(0.0167)
Children in household	−0.0046	0.0075	0.0029	0.0036	−0.0137	−0.0015	−0.00545	−0.0045	−0.0189	−0.0020	−0.0023	−0.0015
	(0.0135)	(0.0117)	(0.0111)	(0.0110)	(0.0129)	(0.0107)	(0.0102)	(0.0099)	(0.0128)	(0.0110)	(0.0106)	(0.0103)
Household head	0.0219	0.0163	0.0224**	0.0225**	0.0157	0.0087	0.0110	0.0108	0.0001	−0.0006	0.0022	0.0024
	(0.0138)	(0.0111)	(0.0107)	(0.0106)	(0.0129)	(0.0099)	(0.0095)	(0.0095)	(0.0139)	(0.0109)	(0.0103)	(0.0102)
ln (1 + Income pre-pandemic)	0.0018	0.0028	0.0031	0.0027	0.0023	0.0029	0.0032*	0.0030*	0.0071***	0.0064***	0.0059***	0.0056***
	(0.0036)	(0.0024)	(0.0020)	(0.0020)	(0.0031)	(0.0020)	(0.0017)	(0.0017)	(0.0018)	(0.0013)	(0.0011)	(0.0010)
Mortgage loans	0.0239				0.0172				0.0302**			
	(0.0146)				(0.0137)				(0.0153)			
Consumer debt		0.0234**				0.0078				0.0275***		
		(0.0097)				(0.0092)				(0.0106)		
Basic goods and services			0.0098**				0.0032				0.0053	
			(0.0047)				(0.0037)				(0.0045)	
Lack of savings				0.0172*				−0.0013				0.0116
				(0.0092)				(0.0089)				(0.0100)
Observations	1,201	1,736	1,919	1,947	1,201	1,736	1,919	1,947	1,201	1,736	1,919	1,947
R-squared	0.030	0.025	0.023	0.021	0.033	0.031	0.027	0.028	0.044	0.026	0.022	0.021
Education level	YES	YES	YES	YES	YES	YES	YES	YES	YES	YES	YES	YES
Region fixed effects	YES	YES	YES	YES	YES	YES	YES	YES	YES	YES	YES	YES

Note: This table reports estimates from a linear probability model (LPM) of the probability of utilizing a range of mental healthcare services against the independent variables. We measure utilization of mental healthcare services with a set of indicator variables that capture a new diagnosis, new treatment, and/or new medication (for a mental health condition), after the start of the pandemic (1 = yes, 0 = no). All regressions control for education and region dummy variables. Heteroskedasticity-robust standard errors are in parentheses. ***, **, and * indicate significance at the 1%, 5%, and 10% levels, respectively.

### Narrowing Down the Channels

We examine conflict within the household as one potential mechanism linking financial distress and mental health outcomes. Specifically, Supplementary Table A5 investigates the relationship between financial distress and increases in the frequency and severity of conflict within the household. Columns (1) to (4) show our financial-distress measures are positively associated with a higher frequency of family conflicts. Columns (5) to (8) show a positive relationship between our measures of financial distress and the intensity of the family conflicts.

In view of our previous results, we augment in Table 4 our baseline regressions reported in Table 2 to control for the frequency of conflicts within the household. The relationship between financial distress and conflicts is highly significant, and the relationship between financial distress and mental health remains highly significant and economically meaningful. Thus, these results confirm financial distress is likely to affect mental well-being both directly and indirectly from a conflict channel.

**TABLE 4:** Mental health, financial distress, and conflicts (Chile, 2020).

	(1)	(2)	(3)	(4)	(5)	(6)	(7)	(8)	(9)	(10)	(11)	(12)
	Poor well-being				Sleep problems				Deterioration			
Unemployment	0.0659*	0.0199	−0.0044	0.0483*	0.1160***	0.0947***	0.0904***	0.1270***	0.0423	0.0572**	0.0552**	0.0871***
	(0.0371)	(0.0292)	(0.0267)	(0.0269)	(0.0373)	(0.0297)	(0.0275)	(0.0271)	(0.0358)	(0.0287)	(0.0266)	(0.0261)
Female	0.0596**	0.0651***	0.0609***	0.0591***	0.1090***	0.1140***	0.1070***	0.1060***	0.0819***	0.0893***	0.0858***	0.0865***
	(0.0279)	(0.0233)	(0.0218)	(0.0221)	(0.0284)	(0.0238)	(0.0223)	(0.0224)	(0.0285)	(0.0236)	(0.0222)	(0.0223)
Age	−0.0046***	−0.0039***	−0.0034***	−0.0039***	−0.0031***	−0.0029***	−0.0028***	−0.0031***	−0.0017*	−0.0018**	−0.0019**	−0.0019**
	(0.0009)	(0.0008)	(0.0007)	(0.0007)	(0.0010)	(0.0008)	(0.0007)	(0.0007)	(0.0010)	(0.0008)	(0.0007)	(0.0008)
Migrant	−0.0636	−0.0832*	−0.0975**	−0.0850**	−0.0855	−0.0908*	−0.0803*	−0.0777*	−0.1550***	−0.1170**	−0.1360***	−0.1210***
	(0.0517)	(0.0432)	(0.0415)	(0.0407)	(0.0548)	(0.0477)	(0.0453)	(0.0440)	(0.0576)	(0.0490)	(0.0472)	(0.0466)
Children in household	−0.0343	−0.0206	−0.0281	−0.0187	0.0281	0.0379	0.0335	0.0372	0.0330	0.0471*	0.0380	0.0361
	(0.0304)	(0.0259)	(0.0243)	(0.0247)	(0.0311)	(0.0261)	(0.0247)	(0.0249)	(0.0302)	(0.0252)	(0.0240)	(0.0240)
Household head	0.0217	0.0349	0.0294	0.0311	−0.0337	−0.0291	−0.0303	−0.0291	−0.0231	−0.0194	−0.0173	−0.0185
	(0.0297)	(0.0248)	(0.0234)	(0.0235)	(0.0303)	(0.0256)	(0.0239)	(0.0240)	(0.0305)	(0.0252)	(0.0239)	(0.0238)
ln (1 + Income pre-pandemic)	−0.0172**	−0.0099*	−0.0073	−0.0132**	−0.0050	0.0009	0.0041	0.0005	−0.0071	−0.0039	−0.0027	−0.0059
	(0.0073)	(0.0057)	(0.0054)	(0.00545)	(0.0070)	(0.0054)	(0.0049)	(0.0049)	(0.0076)	(0.0058)	(0.0054)	(0.0052)
Conflicts at home	0.1970***	0.1880***	0.1650***	0.195***	0.2310***	0.2280***	0.2210***	0.2410***	0.3000***	0.2920***	0.2750***	0.2870***
	(0.0319)	(0.0265)	(0.0249)	(0.0249)	(0.0316)	(0.0262)	(0.0248)	(0.0246)	(0.0294)	(0.0243)	(0.0231)	(0.0228)
Mortgage loans	0.0611*				0.0926***				0.0952***			
	(0.0326)				(0.0326)				(0.0312)			
Consumer debt		0.1190***				0.0932***				0.0987***		
		(0.0239)				(0.0246)				(0.0243)		
Basic goods and services			0.0937***				0.0701***				0.0644***	
			(0.0103)				(0.0105)				(0.0098)	
Lack of savings				0.0517**				0.0584**				0.0958***
				(0.0228)				(0.0234)				(0.0237)
Observations	1,201	1,736	1,919	1,947	1,201	1,736	1,919	1,947	1,201	1,736	1,919	1,947
R-squared	0.096	0.103	0.123	0.084	0.134	0.136	0.151	0.131	0.145	0.148	0.155	0.142
Education level	YES	YES	YES	YES	YES	YES	YES	YES	YES	YES	YES	YES
District fixed effects	YES	YES	YES	YES	YES	YES	YES	YES	YES	YES	YES	YES

Note: This table reports estimates from a linear probability model (LPM) of the probability of experiencing a range of mental health problems against the independent variables. For mental health problems, we create an indicator variable equal to 1 if the individual reports feeling poor or very poor well-being. We also create a dummy variable for sleep problems during the last week. Finally, we include an indicator for well-being deterioration that is equal to 1 if the individual reports that her well-being or mental health has worsened relative to February (before the pandemic). All regressions control for education and region dummy variables. Heteroskedasticity-robust standard errors are in parentheses. ***, **, and * indicate significance at the 1%, 5%, and 10% levels, respectively.

## Discussion

In this paper, we analyze the effects of the COVID-19 pandemic on financial distress and mental health in Chile. We first show how the pandemic affected economic fragility, which in turn increased financial distress (as measured by difficulties in paying for consumer loans, mortgages, or basic needs). We then present evidence that financial distress negatively affects mental health and well-being, using a range of variables and controlling for individual-level characteristics, including pre-pandemic income. Our results are in line with evidence showing that over-indebtedness and the prevalence of depressive symptoms are associated [13], and that poverty is related to psychological distress in Latin America [43]. Our study adds to the growing evidence of the effect of the pandemic in Latin American countries. Many of these countries are characterized by high levels of economic and health inequality, fragile health systems, and high levels of informality and economic fragility [44]. In particular, these features where exacerbated in Chile since the onset of the COVID-19 pandemic, as transmission and mortality rates were found to be related to socioeconomic characteristics [45], there is gender inequality in mental health deterioration and psychological well-being [46], psychological distress is linked to measures of economic uncertainty [47], and increases in food insecurity [48] and domestic violence [49] were reported. In our study we dive deeper into economic vulnerability and several financial problems arising from different household expenditures and household debt, which were previously unexplored in this context. Further, our findings are consistent with evidence from other countries in relation to mental health consequences of the COVID-19 pandemic [4–6, 29, 50, 51].

The pandemic disrupted mental health services in most countries [52]. In our study, although there are increases in new diagnoses and the utilization of medicines for mental health conditions in relation to financial distress, we do not observe an increase in treatments, showing a potential barrier in access to treatment during the pandemic. One reason is that the coverage of mental illness by the Chilean health insurance system is very low, making mental health treatment very expensive for a large proportion of the population, especially for the ones facing financial distress. Another reason are the lockdowns that restricted the mobility of individuals. These results are relevant in a country with high prior levels of household debt [40] and a high pre-pandemic incidence of depressive symptoms [53–55]. On top of this, pre-pandemic rates of mental health care service utilization were already low: in 2017, 2.3% of a nationally representative survey respondents reported having utilized a mental health care service in the previous 3 months [56].

An exploration of the mechanisms linking financial distress and mental health points to a higher frequency of conflicts within the household, consistent with the general finding that domestic violence increased during the pandemic [57] and lockdowns [49, 58–60]. Given the nexus between domestic violence and economic hardship [61] and job loss [62], financial distress is likely to affect mental well-being both directly from the impossibility of meeting or paying financial obligations and indirectly from a conflict channel (i.e., financial distress creates family conflicts that in turn deteriorate mental health). For example, there is a negative relationship between a partner’s fear of job loss [63], or partner’s job loss [64] and own mental health status, showing that the spouse’s financial concerns are an important driver behind the financial distress–mental health nexus. Our results show the need to complement mobility restrictions with specific services to respond to the increase in conflict within households.

Our results highlight the need for new public policies to improve mental health in the short run, for those most vulnerable to the economic impact of the pandemic: younger, female, unemployed individuals. Policies should consider the heterogeneous factors affecting mental health. Our paper also points to the key role of financial education, as a change in financial behavior can improve financial well-being of individuals [65], and financial capability has been shown to be directly related to psychological health, beyond income [66]. Relatedly, a study in the US shows that early in the COVID-19 pandemic the more financially literate people were better able to handle mid-size emergency expenses, indicating knowledge can provide some additional protection during a pandemic [67]. As noted in our study, heavily indebted households suffer directly and indirectly (through conflict) a worsening in their mental health. Financial education, providing individuals with information and tools to alleviate their indebtedness are essential to reduce financial distress and contribute to better mental health and well-being. Recent years have seen an increase in empirical evidence showing that financial education can be effectively provided to large and diverse populations in schools, workplaces and community platforms [68]. Future research should focus on the applicability of these experiences to the local context and their effect of subjective well-being.

### Limitations

An important limitation in our study is that the survey was not designed around a validated instrument to screen for anxiety and depression such as the Patient Health Questionnaire 4 (PHQ-4). We argue that the variables used in our study do a good job in assessing subjective well-being, as modeled by OECD Guidelines on Measuring Subjective Well-being [69]. Another limitation of our study is the representativeness of the sample, which was not constructed from a random sample from the entire population. Our sample covers a higher proportion of professionals and men, and therefore results cannot be extrapolated to the entire population. However, we expect the segments of the population analyzed to be less financially constrained [46]; if this is the case our estimates should be biased towards zero, and are therefore conservative estimates of the overall relationship between financial fragility and mental health. At the time of our study no nationally-representative surveys from the government or other institutions allowed to explore the link between financial fragility and mental health during the pandemic. A final limitation is that the survey data are cross-sectional and limit the possibility of analyzing dynamics, except some of the main outcome variables that are expressed as changes with respect to the pre-pandemic period.

### Conclusion

Understanding the effects on mental health from the pandemic, as well as the effects of the policies implemented, is vital. Relief policies targeting economically fragile and vulnerable individuals are necessary. One key lesson of our findings is that financial assistance in the form of more loans increases debt and may worsen mental health and psychological well-being. Interventions designed with the objective of improving financial education are necessary to increase levels of precautionary savings, build financial resilience, and alleviate the psychological burden of debt in the future.

## References

[1] Bundervoet T Dávalos ME Garcia N. The Short-Term Impacts of COVID-19 on Households in Developing Countries. World Bank (2021). World Bank Policy Research Working Paper 9582. 10.1016/j.worlddev.2022.105844 PMC8823956 35153367

[2] De La Flor L Mujica I Fontenez MB Newhouse D Rodriguez Alas C Sabharwal G Taking Stock of COVID-19 Labor Policy Responses in Developing Countries. Jobs Watch COVID-19. Washington, DC: World Bank (2021). Available from: https://openknowledge.worldbank.org/handle/10986/35331 .

[3] Pierce M Hope H Ford T Hatch S Hotopf M John A Mental Health before and during the COVID-19 Pandemic: A Longitudinal Probability Sample Survey of the UK Population. Lancet Psychiatry (2020) 7(10):883–92. 10.1016/S2215-0366(20)30308-4 32707037 PMC7373389

[4] Proto E Quintana-Domeque C. COVID-19 and Mental Health Deterioration by Ethnicity and Gender in the UK. PLoS ONE (2021) 16(1 January):1–27. 10.1371/journal.pone.0244419 PMC7787387 33406085

[5] Zajacova A Jehn A Stackhouse M Choi KH Denice P Haan M Mental Health and Economic Concerns from March to May during the COVID-19 Pandemic in Canada: Insights from an Analysis of Repeated Cross-Sectional Surveys. SSM Population Health (2020) 12(76me2):100704. 10.1016/j.ssmph.2020.100704 33319028 PMC7723788

[6] Witteveen D Velthorst E. Economic Hardship and Mental Health Complaints during COVID-19. Proc Natl Acad Sci U.S.A (2020) 117(44):27277–84. 10.1073/pnas.2009609117 33046648 PMC7959574

[7] Zheng J Morstead T Sin N Klaiber P Umberson D Kamble S Psychological Distress in North America during COVID-19: The Role of Pandemic-Related Stressors. Soc Sci Med (2021) 270:113687. 10.1016/j.socscimed.2021.113687 33465600 PMC9757831

[8] Frankham C Richardson T Maguire N. Psychological Factors Associated with Financial Hardship and Mental Health: A Systematic Review. Clin Psychol Rev (2020) 77:101832. 10.1016/j.cpr.2020.101832 32088498

[9] Meltzer H Bebbington P Brugha T Farrell M Jenkins R. The Relationship between Personal Debt and Specific Common Mental Disorders. Eur J Public Health (2013) 23(1):108–13. 10.1093/eurpub/cks021 22434207

[10] Butterworth P Olesen SC Leach LS. The Role of Hardship in the Association between Socio-Economic Position and Depression. Aust N Z J Psychiatry (2012) 46(4):364–73. 10.1177/0004867411433215 22508596

[11] Lahelma E Laaksonen M Martikainen P Rahkonen O Sarlio-Lähteenkorva S. Multiple Measures of Socioeconomic Circumstances and Common Mental Disorders. Soc Sci Med (2006) 63(5):1383–99. 10.1016/j.socscimed.2006.03.027 16690186

[12] Sweet E Nandi A Adam EK McDade TW. The High Price of Debt: Household Financial Debt and its Impact on Mental and Physical Health. Soc Sci Med (2013) 91:94–100. 10.1016/j.socscimed.2013.05.009 23849243 PMC3718010

[13] Hojman DA Miranda Á Ruiz-Tagle J. Debt Trajectories and Mental Health. Soc Sci Med (2016) 167:54–62. 10.1016/j.socscimed.2016.08.027 27598550

[14] Riumallo-Herl C Basu S Stuckler D Courtin E Avendano M. Job Loss, Wealth and Depression during the Great Recession in the USA and Europe. Int J Epidemiol (2014) 43(5):1508–17. 10.1093/ije/dyu048 24942142 PMC4190512

[15] Gerardi K Herkenhoff KF Ohanian LE Willen PS. Can't Pay or Won't Pay? Unemployment, Negative Equity, and Strategic Default. Rev Financial Stud (2018) 31(3):1098–131. 10.1093/rfs/hhx115

[16] Elul BR Souleles NS Chomsisengphet S Glennon D Hunt R. What “Triggers” Mortgage Default? Am Econ Rev Pap Proc (2010) 100(2):490. 10.1257/aer.100.2.490

[17] Deng Y Quigley JM Order R Van Order R. Mortgage Terminations, Heterogeneity and the Exercise of Mortgage Options. Econometrica (2000) 68(2):275–307. 10.1111/1468-0262.00110

[18] Sullivan D Wachter Tv.. Job Displacement and Mortality: An Analysis Using Administrative Data. Q J Econ (2009) 124:1265–306. 10.1162/qjec.2009.124.3.1265

[19] Vodopivec MM Laporšek S Stare J Vodopivec MM. The Effects of Unemployment on Health, Hospitalizations, and Mortality – Evidence from Administrative Data. IZA Discussion Paper 14318. Institute for the Study of Labor (IZA) (2021).

[20] Kuhn A Lalive R Zweimüller J. The Public Health Costs of Job Loss. J Health Econ (2009) 28(6):1099–115. 10.1016/j.jhealeco.2009.09.004 19833399

[21] Katz M Bosworth HB Lopes RD Dupre ME Morita F Pereira C A Time-Series Analysis of the Relation between Unemployment Rate and Hospital Admission for Acute Myocardial Infarction and Stroke in Brazil Over More Than a Decade. Int J Cardiol (2016) 224:33–6. 10.1016/j.ijcard.2016.08.309 27611915

[22] Clark AE Oswald AJ. Unhappiness and Unemployment. Econ J (1994) 104(424):648–59. 10.2307/2234639

[23] Winkelmann L Winkelmann R. Why Are the Unemployed So Unhappy? Evidence from Panel Data. Economica (1998) 65(257):1–15. 10.1111/1468-0335.00111

[24] Engelberg J Parsons CA. Worrying about the Stock Market: Evidence from Hospital Admissions. J Finance (2016) 71(3):1227–50. 10.1111/jofi.12386

[25] McInerney M Mellor JM Nicholas LH. Recession Depression: Mental Health Effects of the 2008 Stock Market Crash. J Health Econ (2013) 32(6):1090–104. 10.1016/j.jhealeco.2013.09.002 24113241 PMC3874451

[26] Holmes EA O'Connor RC Perry VH Tracey I Wessely S Arseneault L Multidisciplinary Research Priorities for the COVID-19 Pandemic: A Call for Action for Mental Health Science. Lancet Psychiatry (2020) 7(6):547–60. 10.1016/s2215-0366(20)30168-1 32304649 PMC7159850

[27] Vindegaard N Benros ME. COVID-19 Pandemic and Mental Health Consequences: Systematic Review of the Current Evidence. Brain Behav Immun (2020) 89:531–42. 10.1016/j.bbi.2020.05.048 32485289 PMC7260522

[28] Xiong J Lipsitz O Nasri F Lui LMW Gill H Phan L Impact of COVID-19 Pandemic on Mental Health in the General Population: A Systematic Review. J Affective Disord (2020) 277:55–64. 10.1016/j.jad.2020.08.001 PMC7413844 32799105

[29] Cheng TC Kim S Koh K. The Impact of COVID-19 on Subjective Well-Being: Evidence from Singapore. IZA Discussion Paper 13702. Institute for the Study of Labor (IZA). (2020). Available from: https://ftp.iza.org/dp13702.pdf .

[30] Kola L Kohrt BA Hanlon C Naslund JA Sikander S Balaji M COVID-19 Mental Health Impact and Responses in Low-Income and Middle-Income Countries: Reimagining Global Mental Health. Lancet Psychiatry (2021) 8(6):535–50. 10.1016/s2215-0366(21)00025-0 33639109 PMC9764935

[31] Wolfe M Patel P. Everybody Hurts: Self-Employment, Financial Concerns, Mental Distress, and Well- Being during COVID-19. J Business Venturing Insights (2021) 15:e00231. 10.1016/j.jbvi.2021.e00231

[32] Silverio-Murillo A Hoehn-Velasco L Rodriguez Tirado A Balmori de la Miyar JR. COVID-19 Blues: Lockdowns and Mental Health-Related Google Searches in Latin America. Soc Sci Med (2021) 281:114040. 10.1016/j.socscimed.2021.114040 34144481 PMC9756426

[33] Codagnone C Bogliacino F Gómez C Charris R Montealegre F Liva G Assessing Concerns for the Economic Consequence of the COVID-19 Response and Mental Health Problems Associated with Economic Vulnerability and Negative Economic Shock in Italy, Spain, and the United Kingdom. PLoS ONE (2020) 15(10 October):e0240876–16. 10.1371/journal.pone.0240876 33108374 PMC7591048

[34] OECD. OECD Economic Surveys: Chile 2021 Paris: OECD Publishing (2021). 10.1787/79b39420-en Available from: https://www.oecd-ilibrary.org/economics/oecd-economic-surveys-chile-2021_79b39420-en .

[35] Villalobos Dintrans P Browne J Madero-Cabib I. It Is Not Just Mortality: A Call from Chile for Comprehensive COVID-19 Policy Responses Among Older People. J Gerontol B Psychol Sci Soc Sci (2020) 76:e275. 10.1093/geronb/gbaa092 PMC7454906 32735013

[36] Banco Central de Chile. IMACEC Julio 2020. Nota de prensa (2020). Available from: https://www.bcentral.cl/contenido/-/detalle/imacec-julio-2020 (Accessed May 18, 2022).

[37] Asahi K Undurraga EA Valdés R Wagner R. The Effect of COVID-19 on the Economy: Evidence from an Early Adopter of Localized Lockdowns. J Glob Health (2021) 11:05002. 10.7189/jogh.10.05002 33643635 PMC7897430

[38] INE. Ocupación y desocupación (2021). Available from: https://www.ine.cl/estadisticas/sociales/mercado-laboral/ocupacion-y-desocupacion (Accessed May 18, 2022).

[39] Ministerio del Trabajo y Previsión Social. Presentación de Resultados Encuesta de Protección Social (EPS) VII Ronda (2020). Available from: https://biblioteca.digital.gob.cl/handle/123456789/3854 (Accessed May 18, 2022).

[40] Banco Central de Chile. Cuentas nacionales por sector institucional: Evolución del ahorro, la inversión y el financiamiento sectorial en el segundo trimestre de 2020. (2020). Available from: https://www.bcentral.cl/contenido/-/detalle/cuentas-nacionales-por-sector-institucional-evolucion-del-ahorro-la-inversion-y-el-financiamiento-sectorial-en-el-segundo-trimestre-de-2020 (Accessed May 18, 2022).

[41] Madeira C. The Impact of the COVID Public Policies on the Chilean Households. Appl Econ Lett (2020) 28:1562–5. 10.1080/13504851.2020.1832194

[42] Vida en Pandemia. Facultad de Ciencias Sociales Universidad de Chile. [Internet] (2020). Available from: www.vidaenpandemia.cl (Accessed May 18, 2022).

[43] Rojas M. Poverty and Psychological Distress in Latin America. J Econ Psychol (2011) 32(2):206–17. 10.1016/j.joep.2010.01.014

[44] Benítez MA Velasco C Sequeira AR Henríquez J Menezes FM Paolucci F. Responses to COVID-19 in Five Latin American Countries. Health Pol Technol (2020) 9(4):525–59. 10.1016/j.hlpt.2020.08.014 PMC7451099 32874863

[45] Dintrans PV Castillo C De La Fuente F Maddaleno M. COVID-19 Incidence and Mortality in the Metropolitan Region, Chile: Time, Space, and Structural Factors. PLoS ONE (2021) 16(5 May):1–20. 10.1371/journal.pone.0250707 PMC8101927 33956827

[46] Borrescio-Higa F Valenzuela P. Gender Inequality and Mental Health during the COVID-19 Pandemic. Int J Public Health (2021) 66:8–12. 10.3389/ijph.2021.1604220 PMC8698135 34955701

[47] Duarte F Jiménez-Molina Á. Psychological Distress during the COVID-19 Epidemic in Chile: The Role of Economic Uncertainty. PLOS ONE (2021) 16(11):e0251683. 10.1371/journal.pone.0251683 34731175 PMC8565721

[48] Giacoman C Herrera MS Ayala Arancibia P. Household Food Insecurity Before and during the COVID-19 Pandemic in Chile. Public Health (2021) 198:332–9. 10.1016/j.puhe.2021.07.032 34509858 PMC8428180

[49] Bhalotra S Brito E Clarke D Larroulet P Pino FJ. Dynamic Impacts of Lockdown on Domestic Violence: Evidence from Multiple Policy Shifts in Chile. IZA Discussion Paper 14958. Institute for the Study of Labor (IZA) (2021).

[50] Holingue C Badillo-Goicoechea E Riehm KE Veldhuis CB Thrul J Johnson RM Mental Distress during the COVID-19 Pandemic Among US Adults without a Pre-Existing Mental Health Condition: Findings from American Trend Panel Survey. Prev Med (2020) 139(May):106231. 10.1016/j.ypmed.2020.106231 32758507 PMC7846292

[51] Mata J Wenz A Rettig T Reifenscheid M Möhring K Krieger U Health Behaviors and Mental Health during the COVID-19 Pandemic: A Longitudinal Population-Based Survey in Germany. Soc Sci Med (2021) 287:114333. 10.1016/j.socscimed.2021.114333 34455337 PMC8479385

[52] World Health Organization. The Impact of COVID-19 on Mental, Neurological and Substance Use Services. [Internet] (2020). Available from: https://www.who.int/publications/i/item/978924012455 (Accessed May 18, 2022).

[53] Ministerio de Salud. Encuesta Nacional de Salud 2016-2017 Segunda entrega de resultados (2018). Available from: http://epi.minsal.cl/resultados-encuestas/ (Accessed May 18, 2022).

[54] Ministerio de Salud. Encuesta Nacional de Calidad de Vida y Salud (ENCAVI). [Internet] (2017). Available from: http://epi.minsal.cl/encuesta-encavi-anteriores/ (Accessed May 18, 2022).

[55] Caqueo-Urízar A Urzúa A Aragón-Caqueo D Charles CH El-Khatib Z Otu A Mental Health and the COVID-19 Pandemic in Chile. Psychol Trauma Theor Res Pract Pol (2020) 12(5):521–3. 10.1037/tra0000753 32551750

[56] Ministerio de Desarrollo Social. Situacion de Salud, sintesis de resultados encuesta CASEN. Encuesta de Caracterización Socioeconómica Nacional (CASEN). Gobierno de Chile (2017):1–131.

[57] UN Women. COVID-19 and Ending Violence Against Women and Girls. [Internet]. UN Women (2020). Available from: https://www.unwomen.org/en/digital-library/publications/2020/04/issue-brief-covid-19-and-ending-violence-against-women-and-girls (Accessed May 18, 2022).

[58] Perez-Vincent SM Carreras E Gibbons MA Murphy TE Rossi M. COVID-19 Lockdowns and Domestic Violence: Evidence from Two Studies in Argentina. IDB Technical Note 1956 Inter-American Development Bank. Washington, DC (2020). 10.18235/0002490

[59] Arenas-Arroyo E Fernandez-Kranz D Nollenberger N. Intimate Partner Violence under Forced Cohabitation and Economic Stress: Evidence from the COVID-19 Pandemic. J Public Econ (2021) 194:104350. 10.1016/j.jpubeco.2020.104350 35702337 PMC9186438

[60] Bullinger LR Carr JB Packham A. Covid-19 and Crime: Effects of Stay-At-home Orders on Domestic Violence. Am J Health Econ (2021) 7(3):249–80. 10.1086/713787

[61] Neppl TK Senia JM Donnellan MB. Effects of Economic Hardship: Testing the Family Stress Model over Time. J Fam Psychol (2016) 30(1):12–21. 10.1037/fam0000168 26551658 PMC4742411

[62] Bhalotra S Britto DGC Pinotti P Sampaio B. Job Displacement, Unemployment Benefits and Domestic Violence. IZA Discussion Paper 14543. Institute for the Study of Labor (IZA) (2021).

[63] Bünnings C Kleibrink J Weßling J. Fear of Unemployment and its Effect on the Mental Health of Spouses. Health Econ (2017) 26(1):104–17. 10.1002/hec.3279 26542072

[64] Marcus J. The Effect of Unemployment on the Mental Health of Spouses - Evidence from Plant Closures in Germany. J Health Econ (2013) 32(3):546–58. 10.1016/j.jhealeco.2013.02.004 23524035

[65] de Bruijn EJ Antonides G. Determinants of Financial Worry and Rumination. J Econ Psychol (2020) 76:102233. 10.1016/j.joep.2019.102233

[66] Taylor MP Jenkins SP Sacker A. Financial Capability and Psychological Health. J Econ Psychol (2011) 32(5):710–23. 10.1016/j.joep.2011.05.006

[67] Clark RL Lusardi A Mitchell OS. Financial Fragility during the COVID-19 Pandemic. AEA Pap Proc (2021) 111:292–6. 10.1257/pandp.20211000

[68] Lusardi A. Financial Literacy and the Need for Financial Education: Evidence and Implications. Swiss J Econ Stat (2019) 155(1):1–8. 10.1186/s41937-019-0027-5

[69] OECD. OECD Guidelines on Measuring Subjective Well-Being. [Internet]. Paris: OECD Publishing (2013). Available from: https://www.oecd-ilibrary.org/economics/oecd-guidelines-on-measuring-subjective-well-being_9789264191655-en . 24600748

