# Supplementary material for: Financial Distress and Psychological Well-Being During the COVID-19 Pandemic
Source: Int J Public Health. 2022 Aug 25;67:1604591. doi: 10.3389/ijph.2022.1604591 (PMC9453756; doi:10.3389/ijph.2022.1604591)
Supplement: Supplementary file 1 [file DataSheet1.docx]

**Supplementary Material**

1. **Robustness checks**

We have shown a strong relationship between financial distress and mental health. However, potential endogeneity or reverse causality in our OLS estimates may produce biased estimates of the impact of financial distress on mental well-being. For example, financial distress can affect mental well-being, which may make acting to address a difficult financial situation harder. To mitigate concerns associated with endogeneity biases, we re-estimate our baseline regressions by using instrumental variables (IV) techniques. Our approach employs a synthetic instrument for each of our measures of financial distress, by calculating the average rate of the financial distress by gender-age-district. For each gender-age-district we compute the average rate of financial distress (for all our 4 measures) and use these synthetic variables as instruments for the actual financial distress variables.

Table A.6 in this appendix reports the results from the second stage of our IV regressions. Our main results remain qualitatively unchanged. Table A.7 reports the results from our first stage regressions. As reported by the partial R-squared of excluded instruments and F-tests, the partial correlation between the instruments and the endogenous variables are not zero.

1. **Appendix Tables**

**Table A.1: Descriptive Statistics - Outcome Variables (Chile, 2020)**

Note: This table reports descriptive statistics (mean, standard deviation, minimum value, and maximum value) for the dummy variables associated with financial distress, mental health, emotions, healthcare, and conflict.

**Table A.2: Descriptive Statistics - Independent Variables (Chile, 2020)**

Note: This table reports descriptive statistics (mean, standard deviation, minimum value, and maximum value) for the independent variables. Control variables include the following: an indicator for female, age, an indicator for migrant status, an indicator for the presence of young children (under the age of 12) in the household, an indicator for household head, pre-pandemic household income (expressed in natural logarithm), an indicator for unemployment and an indicator for college degree. In the regressions we employ education-achievement indicators (complete or incomplete primary, secondary, technical, bachelor’s, and master’s level or more).

**Table A.3: Mental Health and Individual Level Characteristics (Chile, 2020)**

Note: This table reports estimates from a linear probability model (LPM) of the probability of experiencing a range of mental health problems against the independent variables. For mental health problems, we use an indicator variable equal to 1 if the individual reports feeling poor or very poor well-being. We also include a dummy variable for sleeping problems during the last week. Finally, we include an indicator for well-being deterioration that is equal to 1 if the individual reports that her well-being or mental health has worsened relative to February (before the pandemic). All regressions control for education and region dummy variables. Heteroskedasticity-robust standard errors are in parentheses. ***, **, and * indicate significance at the 1%, 5%, and 10% levels, respectively.

**Table A.4: Emotions and Financial Distress (Chile, 2020)**

Note: This table reports estimates from a linear probability model (LPM) of the probability of experiencing a range of negative feelings against the independent variables. Measures of negative emotions include distress, frustration, worry, and restlessness. We use a dichotomous variable for each measure, that is equal to 1 if the individual reports experiencing the abovementioned condition or feeling frequently or very frequently, and 0 otherwise. All regressions control for a comprehensive set of control variables, including dummy variables by region, we only report our coefficients of interest for space considerations. Heteroskedasticity-robust standard errors are in parentheses. ***, **, and * indicate significance at the 1%, 5%, and 10% levels, respectively.

**Table A.5: Conflicts and Financial Distress (Chile, 2020)**

Note: This table reports estimates from a linear probability model (LPM) of the probability of experiencing a higher frequency and an intensity of conflicts within the household against the independent variables. All regressions control for region dummy variables. Heteroskedasticity-robust standard errors are in parentheses. ***, **, and * indicate significance at the 1%, 5% and 10% levels, respectively.

**Table A.6: Mental Health and Financial Distress – IV (Chile, 2020)**

Note: This table reports estimates from an Instrumental Variables (IV) model of the probability of experiencing a range of mental health problems against the independent. For mental health problems, we create an indicator variable equal to 1 if the individual reports feeling poor or very poor well-being. We also create a dummy variable for sleeping problems during the last week. Finally, we include an indicator for well-being deterioration that is equal to 1 if the individual reports that her well-being or mental health has worsened relative to February (before the pandemic). The models employ a synthetic instrument for each of our measures of financial distress, by calculating the average rate of the financial distress by gender-age-district. All regressions control for education and region dummy variables. Heteroskedasticity-robust standard errors are in parentheses. ***, **, and * indicate significance at the 1%, 5%, and 10% levels, respectively.

**Table A.7: First Stage IV (Chile, 2020)**

Note: This table reports first stage estimates from a linear probability model (LPM) of the probability of experiencing a range of financial problems against the independent variables. The measures of financial distress include an indicator variable for difficulty paying mortgage loans and an indicator variable for difficulty paying consumer debt. We also construct a measure from 0 to 4 regarding whether the individual has problems paying for basic goods and services: (1) basic goods, (2) medicine, (3) rent, and/or (4) school. Finally, we use an indicator variable for lack of savings, where savings are measured as the number of months the respondent believes that basic expenses can be afforded with savings if the main income source is lost. All regressions control for region dummy variables. Heteroskedasticity-robust standard errors are in parentheses. ***, **, and * indicate significance at the 1%, 5%, and 10% levels, respectively.
